# Observations on the Functions of the Spleen and Other Bodies in the Animal Economy, the Uses of Which Are Not Clearly Understood

**Published:** 1832-06

**Authors:** 


FUNCTIONS OF THE SPLEEN, &c.

***Observations on the Functions of the Spleen and other Bodies in the Animal Economy, the Uses of which are not clearly understood.*** By R. H., A.B., M.D., Member of the Royal College of Surgeons, London.

THAT nature has made nothing in vain, is a maxim in which I fully concur. What then are the uses of the Spleen, Thyroid and Thymus Bodies, Renal Capsules, and other organs in the animal structure, whose functions, if not totally unknown, are at most only guessed at? But, surely, in the human body, which is the masterpiece of creation, these bodies must be intended to fulfil some useful purposes! What are they then? In this little paper, I have not the presumption to pretend to divulge the secret of the purposes for which these organs are given us. A secret which has been hidden for so many generations, and which more learned and much wiser heads than my own have not been able to discover, may well baffle all my endeavours: but certainly, with many others, I may be permitted to hazard conjectures, and leave it to the more learned to judge how near to, or how remote I may be from, the truth.

I shall, in the first place, offer some general remarks as to the use of these organs collectively, and then say a few words of each separately. The organs to which I shall principally confine my observations are the spleen, thymus and thyroid bodies, and renal capsules. So much has already been said and written respecting the liver and its uses, that I shall pass it over in silence, or nearly so. Indeed, the part which the liver acts in the animal economy appears to be better understood than that of any of the other organs which I have named; yet I am by no means satisfied that its (the liver's) real, or at least full, uses are thoroughly known: in fact, the variety of jarring opinions respecting it confirm this opinion. As to myself, I imagine the liver to be an organ more for the purpose of depuration than for the secretion of a fluid absolutely necessary for the process of digestion.

The term gland, as applied to the liver, may be correct enough; but I regard it as highly incorrect when applied to the other bodies I am about to treat of, these being organs (as I suppose) neither of secretion nor depuration. The bodies above named I regard as exclusively appertaining to the circulating system, chiefly acting as moderators to the currents of blood going to certain delicate and vital organs; or (to use a mechanic's expression) acting as safety valves to these important organs. This use I especially attribute to the spleen and thyroid gland or body: probably the thymus gland* and renal capsules serve the same good office, more particularly after the birth of the individual. These last appear to have other uses, which I shall touch on when speaking of them.

It is not my intention here to enter into or discuss the merits of the different opinions held by others on this subject: I mean, as much as possible, to confine myself to my own conjectures. The spleen, from its magnitude, may claim our first attention: this is a large, somewhat oval, fleshy organ, placed nearly in the centre of the body, and attached to the great *cul-de-sac* of the stomach; it is generally thought to be subservient to the stomach and liver, aiding in the formation of gastric juice and bile.

That the spleen is subservient to the stomach I verily believe, but not in aiding it to supply its gastric juice, nor as a reservoir of blood, from which it (the stomach) can draw on at pleasure, or when secreting its necessary fluid; but that the spleen is subservient to the liver, or directly assists it in the formation of bile, I have considerable doubts.

The use that I attribute to the spleen is, that, situated amongst delicate and vital organs, (the stomach, intestines, and liver, &c.;) it serves as a safety valve to preserve these important parts from arterial plethora, by regulating the current of blood under such circumstances as would be likely to induce that accident.

To make myself more clearly understood, suppose, in some of the more violent actions of the body, when, from the strong contractions of the muscles, the blood is forced from amongst them to the interior and less resisting parts; or, again, in severe rigors, when blood is driven from the surface of the body to the interior of it, or in obstruction to the circulation of the blood in the lungs or great vessels, or in high arterial excitement from any cause whatever: under these or similar circumstances, I say, the stomach, duodenum,

* See the Review of Sir ASTLEY COOPER's work on the Thymus Gland, in the present number.—ED.

perhaps the liver, and other neighbouring delicate parts, would be in great danger of hemorrhagies, or of being inflamed by a sudden and unusual flow of blood to them, if that flow was not counteracted by the moderating power of the spleen, which unharmed receives the impetus of the blood.

When we consider the texture of the mucous membrane of the stomach, intestines, &c., its very great vascularity, the extreme delicacy and superficial situation of vessels giving this vascularity: when we consider, I say, these circumstances, we may well be astonished that hemorrhage so seldom (comparatively speaking) occurs in these important organs during the accidents and vicissitudes to which the human body is subject. Is it not then reasonable to suppose that there must exist some guardian, some protecting, power, to watch over and defend those parts? Now, the situation, relations, and structure, of the spleen, as likewise the source from whence it receives its blood, all declare that organ to be this guardian, this protecting, power.

First, the situation and relations of the spleen. It is situated in the abdomen, amongst and in close relation with those organs it appears destined to protect. Being placed in the abdomen, it (the spleen) may swell, in case of emergency, to the utmost of its extent, without injuring or materially interfering with the functions of the vital organs placed around it; for in this case (as in the descent of diaphragm) it is the relaxation of the parietes of the abdomen that makes room for its enlargement. The structure of the spleen is precisely such as to fit it for the function here attributed to it: it is cellular and spongy, resembling, if not altogether similar in structure to, the body of the penis, thereby readily admitting a sudden influx of blood, and also permitting as ready an exit to that fluid when circulation through the liver is uninterrupted; but if interrupted, which consequently retards the circulation through the spleen, then the spleen is capable of swelling out, like the penis, to a considerable magnitude, thus accommodating a large quantity of blood till this sudden emergency is gone by.

That the spleen is capable of this sudden enlargement, or erection, is satisfactorily proved by the simple but cruel experiment of strangling a dog, having previously exposed that body. Almost immediately on the circulation being impeded, from want of air to the lungs, the spleen will be seen to swell out to an enormous size. Now, for what purpose is the spleen endowed with this erectile property, if not for the use herein stated? The penis possesses the same property, but its use in that organ is well known: here my first observation has its weight, "nature does nothing in vain."

The question may here be asked, if such be the use of the spleen, how comes it, for what purpose does it empty its blood into the venæ portæ? thus distributing it in common with the blood from the intestinal canal into the *parenchyma* of the liver? Why not send its blood directly into the cava ascendens, if it be of no service either to the liver or in the formation of bile? It is not my intention, as I before observed, at present to enter into the merits of the different opinions respecting the use of the spleen. At some future period, when I shall have made certain investigations and experiments in which I am at present engaged, I shall be able more fully to enter on this subject. However, in answer to the above question, I shall here offer a few observations relative to the connexion between the spleen and liver.

I am of opinion that the liver may, to a certain degree, and under certain circumstances, become an auxiliary to the spleen, receiving a certain portion of blood from that organ, when the circulation in the cava ascendens is by any accident impeded. I am led to this opinion by the structure of the liver, which appears to favor its distention, though perhaps in a very limited degree. However this may be, I can readily conceive that, under all circumstances, the blood from the spleen is certain of finding a readier passage into the cava, through the medium of the liver, than if the splenic veins emptied themselves directly into that leading channel; for, suppose the latter to be the order of things, then every slight obstruction to the regular flow of blood through the cava would necessarily very much impede the return of that fluid from the spleen; but, as the case is, the splenetic vein empties itself into the vena portæ, which, by dividing itself into innumerable lesser vessels, finds a ready entrance into the substance of the liver, and thereby a more certain or less interrupted passage for its blood into the general circulation. Nor do I think it unreasonable to suppose that the liver is endowed with a certain considerable power of propelling its blood into the cava, at least a greater power than could be expected to reside in a vein going directly from the spleen into that great vessel. Indeed, when we consider how important it is to the animal economy that that large stream of blood coming from the intestinal canal, and charged with the fresh supplies of nutriment for the body, should uninterruptedly, and with all expedition, pass through the liver into the great circulation, we are led, I think, even without direct proof, to admit that such a propelling power exists in that organ. An indirect proof of this is offered (to me at least,) by the long route which the thoracic duct makes to reach the descending cava, or, more correctly, the left subclavian vein which empties itself into that vessel; as by this means it (the thoracic duct) ensures to itself a freer and less interrupted discharge for its nutritious fluid into the general circulation, than if it emptied itself into the cava ascendens. Such circumstances, though apparently trifling, often argue a great deal. The spleen is supposed by many to concoct, or render the blood that passes through it fit for the yielding of bile to the liver: now this I think is very improbable, considering the structure of the spleen, which does not appear to be glandular, or in the least calculated for effecting any particular alteration in the blood; but, like the penis after erection, delivers up that fluid very little changed from the state in which it was received. But the blood from the spleen may be essential to the liver, in order to supply a sufficient quantum of bile? Even this I feel disposed to question; for I am led to regard the bile more as a secreted excretion (like urine) than as a secretion actually necessary to the process of digestion: as an excretion, I would say, from that heterogeneous mass of fluids taken up by the veins from the stomach and intestines; and the liver I regard as the organ of depuration to this crude mixture, in like manner as the kidneys are to the general mass of blood.

It is now well known that the total of alimentary matter which passes into the circulation is absorbed or taken up from the alimentary canal by two sets of vessels, namely, the lacteals and veins. Now, it appears to me that these two sets of vessels do not take up from the general mass of nutriment similar fluids: the lacteals absorb the more pure, the veins the more gross. The lacteals, as it were, possessing more judgment than the veins, only drink up such of the fluid from the great mass of nutritive matter as may mingle in the general circulation, without first undergoing the process of depuration: but the veins gorge themselves with whatever comes in their way; and, consequently, take up a vast deal of matter which could not enter the general mass of blood without very inconvenient, or perhaps quickly fatal, consequences. To purify, then, or deprive this mass of blood and other fluids coming from the intestinal canal of any injurious matter, is *(selon moi)* the great function of the liver; thereby rendering them (like the fluids absorbed by the lacteals) fit to mix with the general circulation. Certainly, admitting this theory, the blood from the spleen may both favor the secretion of bile and be of service to the liver itself, by diluting the crude fluid coming directly from the alimentary canal; thus subjecting its particles to the more complete action of the liver, and likewise in a measure protecting that organ from any acrid or baneful qualities which may exist in the unfiltered fluid itself.

However this may be, I am of opinion that the principal, if not the only service rendered to the liver by the spleen, is the preservation of it, in like manner as it (the spleen) preserves the stomach and duodenum from arterial congestion under the circumstances already alluded to. This may appear rather paradoxical, viewing the fact I have just now observed, viz. that of the spleen emptying its blood into the liver, and thus sometimes, of course, assisting to gorge it with that fluid. But we must consider that this congestion, and that arising from an undue or sudden rush of blood into its arteries, are very different as to their probable consequences. The source from whence the spleen draws its blood seems also to favor my view of its use, being the same as that from which the stomach, liver, and upper portion of intestines draw their blood. The cæliac trunk, which is very short but thick, divides into three branches, one for the liver, one for the stomach and duodenum, and one for the spleen. Now, this last is generally the most considerable of the three, and appears to be the continuation of the mother trunk. This circumstance concurs with the others already mentioned (structure of the spleen, &c.) to give vent or a free passage to blood from the cæliac axis in disturbed circulation, or when that fluid rushes with greater force and in larger quantity than usual into that vessel, and by this free vent preserves the gastric and hepatic arteries from an impetus of blood which might prove injurious to the important organs they supply. Again, the stomach receives several of its arteries from the splenic: this is a wise provision of nature, as, under the circumstances above mentioned, the splenic artery unloads itself into the spleen, (as the blood will necessarily flow to where the passage is freest,) by means of the particular branches to that body, thereby leaving those branches which supply the stomach comparatively free from congestion.

If I look into the pathology of the spleen, I shall there also find matter to strengthen my views on this subject: for instance, from long-continued ague, or in persons who have been subject to frequent attacks of that disease, the spleen not unfrequently has been found very much enlarged. Whence is this? In no disease, perhaps, is the circulating system so disturbed as in intermittents: the blood during the rigors is forcibly driven in on internal parts from the surface of the body; the consequence of which is, that the spleen, in long-continued cases, constantly receiving the impetus of it, and being swollen by it, its cells, at length, by frequent distentions, become permanently enlarged; consequently, the mass of the spleen itself. The inference from this is evident: if the spleen were not placed in the interior of the body, amongst the delicate organs which surround it, and likewise adapted, after the manner I have pointed out, to receive any extraordinary impetus of blood—quere: what evil consequences might then ensue to these delicate and vital organs? From what I have now said, it may be seen that the principal use that I attribute to the spleen is to watch over and protect the stomach, duodenum, and liver, from the evils or accidents which might arise to them from sudden and untoward disturbances of the circulation.

Having said so much relative to the functions of the spleen, I shall be able in a few words to convey my idea of the uses of the other bodies before named. I shall next offer some observations on the thyroid body, or gland, as it is termed; as I conceive that it performs the same good office to the brain and delicate organs situated in the larynx (more especially to the brain,) that the spleen does to the stomach and other important parts connected with it. The thyroid body is situated on the fore part of the neck, covering part of the thyroid cartilages and the superior rings of the trachea: its substance, when cut into, has certainly more of a glandular appearance than the spleen, but, on close examination, it will, I think, be found not to differ very materially from that large body in its structure. The situation, structure, sources of blood, and other circumstances relating to the thyroid, all concur to strengthen my opinion, that its use is really that which I have above attributed to it, namely, to act as a safety valve to the brain and delicate organs in the larynx. The thyroid body is placed on the centre of the fore part of the neck, as the most convenient situation for receiving blood from the right and left carotids, (the principal sources of blood to the brain,) being equidistant from both these vessels. It is placed quite superficially, being in great part covered but by integuments, that the circulation through it might be as little impeded as possible by pressure on it from other parts, which undoubtedly would be the case had its situation been deeper in the neck: so much for position. The structure of the thyroid appears to me to be cellular and spongy, and of a texture every way best calculated to favor a ready transit of blood from its arteries into its numerous veins, and thence into the great circulation. Indeed, simply to act as a medium to afford this free passage of blood appears to me to be the sole use of the thyroid, thereby performing the essential function allotted to it by the Allwise.

The principal arteries of the thyroid arise at or near the bifurcation of the common carotids, or where they divide into internal and external; sometimes they arise from the internal carotids, but are generally the first branches given off by the external. The thyroid arteries are very considerable, and, admitting the blood to pass freely from the carotids into the thyroid body, are well calculated to break down and diminish any unusual impetus of that fluid in the internal carotids, or rather to ward off any such impetus from them, and in this manner preserve the brain from perhaps the fatal effects of a surcharged circulation, which disease or accidents might give rise to.

Even pathology, I think, will hold out some support to my idea of the use of the thyroid. During a residence of some time on the Pyrennean mountains, I observed very many individuals afflicted with goitre, (a swelling of the thyroid body,) among whom several were idiots. This circumstance attracted my attention, and I imagined it argued some direct connexion between the thyroid body and the brain. Upon investigation, I found it was no uncommon occurrence for those persons who were afflicted with this disease (goitre) to a considerable degree from childhood, to lack their reasoning powers. To account for this pathological phenomenon, I decided in my own mind that the thyroid body, when much enlarged, (sometimes it more than doubles the bulk of the head in magnitude,) abstracts such a vast quantity of blood from the natural supplies to the head, that, when children are the subjects of it, the full development of the brain cannot take place; and hence a deficiency in the functions of that most important organ. In fact, the above circumstance it was that first led me to inquire into the subject of this paper, and, following up my ideas and arguments, I arrived at the conclusions herein stated.

Supposing the uses here assigned to the spleen and thyroid to be the true ones, the difference of magnitude of these two bodies will immediately strike our senses, and make us inquire why the thyroid, which protects such a large, delicate, and vital organ as is the brain, should be so diminutive in comparison with the spleen, which protects less delicate parts. The spleen is likewise endowed with a protecting property which the thyroid does not appear to possess: I mean, its power of erection or enlargement. How is this? I think I can explain this extraordinary difference in the two bodies, and shew that it is quite compatible with the functions assigned them in this paper.

In the first place, the brain being a long way removed from the centre of circulation, the impetus of blood must be considerably lessened before it reaches that organ: then the head, in the human species, is always more or less in an erect posture, a circumstance that must tend to impede the flow of blood to it, the gravity of that fluid acting to this end; again, a great portion of the impetus of blood in the common carotid is lost by its division into internal and external; but the two following circumstances are those which may best reconcile us to the diminutive size of the thyroid in comparison with the spleen, considering its (the thyroid's) important office. Nature, well aware of the delicacy of her work when she formed the grand centre of sensation, has, by a beautiful contrivance at the entrance into the skull, placed another safeguard over the brain: I allude to the tortuous manner in which the carotid arteries enter the cranium, and likewise the union and subsequent division of the vertebral arteries, &c. Indeed, I think that this circumstance gives additional weight to my doctrine, as it affords an evident and admitted proof that Providence foresaw the necessity of placing safeguards to watch over and protect her more delicate and important structures or organs from the baneful influence of an oppressive or a too-violent circulation.

But this is not the only evident instance, even in the human body, of a protecting contrivance against arterial plethora. I regard as a still more beautiful one the manner in which the mesenteric arteries are distributed in the mesentery previous to their entering the intestines. Here we find the arteries uniting, then separating, and upholding such communications one with another as to form a complete tissue of network in the substance of the mesentery. Now, for what purpose can this condition of the blood-vessels serve, if not to preserve the intestines from any unusual impetus of blood which accident or ought else may promote; and to admit of this peculiar distribution of the arteries going to the intestines, appears to be one of the principal uses of the mesentery, at least, of its very considerable width. In the heads of some quadrupeds, the contrivance of nature for this end is very remarkable. In the last place, the veins of the thyroid body, discharging their contents into the jugular or subclavian veins, thence into the cava descendens, are not so subject to meet with obstruction as veins from the spleen, or those which discharge their blood into the cava ascendens, the column of blood, by its gravity, naturally increasing any obstruction which may chance to impede the regular ascent of fluid in that vessel towards the heart: whereas, just the contrary is the case in the cava descendens: in it, the weight of the column of blood must materially assist the descent of that fluid towards the heart; and to this latter circumstance principally I attribute the great magnitude of the spleen over the thyroid body, as also the peculiar property it (the spleen) possesses, that of erectibility, or swelling out, as occasion may require. By these properties the spleen is enabled, in cases of emergency, as when the free return of blood to the heart through the cava ascendens is impeded, to receive a considerable quantity of that fluid, (of which it can disgorge itself at the first convenient opportunity,) and thus protect the important parts intrusted to its *surveillance* from the accidents attendant on sudden arterial plethora. Besides, if the thyroid were larger than it at present is in its natural state, it would destroy the symmetry of the neck; whereas, in its present condition, it rather adds to the beauty of that portion of the body, by concealing the not very elegant angles about the larynx.

I do not think that the brain, already possessing a safeguard in the manner in which its vessels reach it, is a circumstance that tells against the thyroid as acting in the same capacity. The more valuable our treasure, the more care and protection it requires. Even as matters stand, how often do we see the fatal effects of arterial congestion on the brain?

When I consider the very close vascular connexion between the thymus and the organs contained in the larynx, (the epiglottis and the organs of voice,) I think it by no means improbable but that these delicate parts are materially benefited by the thymus, in the same manner as is the brain.

To conclude my observations on the spleen, I shall briefly observe, that since we find nature so provident in placing certain contrivances for the protection of some of the delicate and important organs in the animal structure against arterial congestion: as to the brain and intestines, (for that the peculiar disposition of the arteries in the mesentery is for that purpose, scarcely, I think, admits of a doubt;) is it not reasonable, I say, to suppose that nature, finding her thus provident to some organs, could not overlook others equally important, as the stomach, duodenum, and liver? And surely the stomach, the most delicate and essential portion of the alimentary canal, requires as much as, if not more than, any other part of that canal, some similar provision for its security; some safety valve to guard it against any irregularities which may arise in the circulation: the spleen, I maintain, is provided by nature for this purpose.

The lungs, during the fœtal period, or previous to birth, lie in the thorax in a state of perfect collapse and passiveness, requiring no more arterial blood than is actually necessary for their existence and development; but, on the birth of the infant, the lungs become inflated, and their vital functions are called into immediate action; then it is that a greater supply of arterial blood is required by them (the lungs) to give them the vigour necessary for the due performance of their functions; and, as the development of the lungs now more rapidly goes on, an additional supply of the nutritious fluid is, of course, called for by them. Now, my idea of the thymus gland, and its use, is, that it is a body appointed by nature to hold in trust a certain portion of blood for the lungs during their minority, or before birth, which trust is to be delivered up immediately on their wards (the lungs) becoming of age, or after the birth of the infant. The thymus, then, no longer having this important duty to perform, wearies of life, and dwindles away: but I am of opinion that, even diminutive as the thymus becomes, it still continues to serve the lungs, acting the part towards them through life that the thyroid body and spleen act *(selon moi)* towards the brain and stomach, &c. This figurative language, in a writing like this, will, I know, be deemed objectionable by some; but I use it finding it the clearest and most expeditious way for conveying my ideas on the subject I treat of. There is another important office which the thymus appears to hold during the fœtal period, which is that of filling up to a certain extent the cavity, as it is improperly termed, of the thorax; so that, after birth, it (the thymus) dwindling away, leaves a more considerable space for the development and inflation of the lungs. The thymus may likewise be serviceable to other of the thoracic viscera, in the same manner as it serves the lungs.

The renal capsules I regard as bodies performing precisely the same services to the kidneys as the thymus to the lungs, both previous to and after birth, except that of retaining space for the more full development of the kidneys, which, for obvious reasons, would be unnecessary in the abdomen, even did those glands considerably increase in volume after birth.

I shall now offer a few observations relative to those reservoirs of blood, (if I may so term the thymus and the renal capsules,) which I look on as a necessary provision for those very essential parts they are intended to supply.

The kidneys, (I shall at present confine my remarks to these bodies and their capsules,) like the lungs, require no more blood during their fœtal state than is sufficient for their development and support. Nay, did they receive more of this fluid than necessary for the above purposes, the consequences would, in all probability, be very injurious, if not fatal, to the contents of the womb; as the kidneys might then be stimulated to act. What, in such a case, would become of the urine? what the consequences of its distillation? But, after birth, the capsules, by a law of nature, close their sluices, and thus force the current of their blood to the kidneys, thereby stimulating them to act, and at the same time supplying a sufficient quantity of the general mass of blood to undergo the purifying action of those organs. And that this really is the case I am led to believe, by considering that the renal capsules, immediately after birth, begin to diminish; and we all know that a current of water, or other fluid, when it finds the channel blocked up in which it has been in the habit of running, will, by a law of hydrostatics, take possession of the first channel it can find. Now this is precisely the case in point: the stream of blood to the renal capsules, finding its channel blocked up, takes possession of that leading to the kidneys, which lies immediately next it; and, that this may the more effectually take place, the renal capsules dwindle away, or shut up their floodgates gradually, according as their fluid is accommodated with a passage by the kidneys.

Should the capsules at once close their sluices, the object for which nature *(selon moi)* formed them would not be attained, as a considerable portion of their blood would, in such case, be forced back into the general circulation.

The renal capsules receiving a great proportion of their blood from the emulgent arteries, ensures the more certain flow of that fluid to the kidneys, when they (the capsules) refuse to receive it, or have no further occasion for it. Again, had not all-wise Providence had recourse to this, or some such, contrivance for ensuring an additional supply of blood to the kidneys after birth, they (the kidneys) should either be content with the portion originally allotted to them or else procure the necessary supplies at the expense of, or detriment to, some other part or organ; seeing that every part of the body has a just proportion of blood allotted to it, according to its wants and functions. That fluid, therefore, can be but ill spared from one part to supply the casual wants of another. These observations, though here confined to the renal capsules and kidneys, may be referred to the thymus body and lungs, being quite applicable to them. I am likewise of opinion that the renal capsules, during respiratory life, perform the same good office to the kidneys as I

suppose the spleen and thyroid do to the stomach, brain, &c. I have already made the same remark respecting the thymus and lungs. How the thymus gland and renal capsules decrease after birth, I shall not here stop to inquire: suffice it to observe, that they do so by an evident law of nature, in like manner as the foramen ovale of the heart and ductus arteriosus, &c. close up, since their remaining open would be detrimental to the animal economy.

I have now offered to the consideration of the faculty my opinions respecting those bodies in the animal economy, the uses of which are but imperfectly understood: I do not pretend to say that I have fully proved any thing; but this I do think, that I have offered some good circumstantial evidence in support of my views of the subject here treated of; and such evidence is sometimes sufficiently conclusive, and may here perhaps prove so, to satisfy some at least, that the opinions herein offered are not altogether erroneous. If, however, I have thrown any light on the obscure subject treated of in this paper, my end, if not completely attained, will certainly be far advanced.

---

